# Mapping QTL associated with *Verticillium dahliae* resistance in the cultivated strawberry (*Fragaria × ananassa*)

**DOI:** 10.1038/hortres.2015.9

**Published:** 2015-03-11

**Authors:** L Antanaviciute, N Šurbanovski, N Harrison, K J McLeary, D W Simpson, F Wilson, D J Sargent, R J Harrison

**Affiliations:** 1 East Malling Research, New Road, East Malling, Kent ME19 6BJ, UK; 2 Research and Innovation Centre, Fondazione Edmund Mach, Via E. Mach 1, 38010 San Michele all’Adige (TN), Italy

## Abstract

A biparental cross of octoploid strawberry segregating for resistance to *Verticillium dahliae*, the causative agent of Verticillium wilt, was screened under field conditions for three seasons. Average wilt scores were significantly associated with multiple QTL, which were mostly significant across all years. Markers significantly associated with the traits were used to screen material with known wilt resistance and susceptibility phenotypes. A clear and statistically significant relationship was observed between resistant, tolerant and susceptible material and the total number of markers present in the different resistance classes. In field situations resistance QTL appear to behave in an additive manner. These markers are abundant in the cultivated strawberry germplasm indicating that, despite the large number of markers, clear genetic gain is possible through marker-assisted breeding.

## Introduction

The vascular wilt pathogen *Verticillium dahliae* is a primary pathogen of the cultivated strawberry *Fragaria × ananassa*, although the pathogen has a far wider host range; up to 200 different dicotyledonous plant species are reported to become infected by *V. dahliae* including artichoke, eggplant, pepper, potato and tomato.^1^ There are some reports of host specialisation measured by levels of virulence on susceptible hosts, indicating that the pathogen population is heterogeneous for host-specific virulence factors.^2^

*Verticillium dahliae*, in common with other *Verticillium* spp. is a soil-borne pathogen, which forms long lasting multicellular structures called microsclerotia. Microsclerotia, in the absence of excess carbon and nitrogen in the soil (from root exudates) are dormant, germinating only when nutrient concentrations rise. It is thought that only a portion of cells in the microsclerotia germinate with any single activation event, leading to the long-term survival of the structure. Invasion has been reported to occur by entering at the root tip and at the site of lateral root branching;^3^ these are sites at which the root endodermis is not fully formed. The endodermis is the layer of cells surrounding the pericycle and the vascular cylinder (working from the outside of the root inwards).^4^ Once the vascular cylinder has been penetrated, *V. dahliae* then begins to form conidia, which travel via passive transport acropetally through the xylem system. Following sporulation in the root, stem vessels are subsequently colonized and fungal proliferation occurs in a cyclical manner, which may be driven by plant defence responses.^5^ Once colonized and sufficient fungal biomass has accumulated, the plant begins to wilt and symptoms of necrosis are observed. During this stage the pathogen enters a saprophytic stage of its life cycle, producing large numbers of microsclerotia from decaying tissue, which are released into the soil for infection in subsequent years. *V. dahliae* is a monocyclic pathogen, with only a single round of disease and inoculum production occurring in a growing season, though the pathogen can overwinter within perennial hosts, meaning that the whole infection cycle need not be repeated in subsequent years.^6^

Resistance to *V. dahliae* has been reported in several systems, including mint,^7^ potato,^8^ cotton^9,10^ and tomato.^11^ In the case of tomato, recent work has identified a pathogen effector gene, named *Ave1*, the protein product of which is responsible for activation of resistance against race 1 strains of *V. dahliae*. In the host this is mediated by the host receptor-like kinase gene *Ve1*.^12^ Follow-on work has shown that the resistance to *Ave1* varies among Solenaceous species, *Nicotiana glutinosa* has a homologue of *Ve1* that is active against race 1 *V. dahliae* isolates, but other species such as *Nicotiana benthaminiana*, not only lack *Ve1* homologues, but fail to activate the hypersensitive response in the presence of co-expressed Ave1 and Ve1 by agrobacterium transient expression.^13^ The gene for gene relationship between *Ave1* and *Ve1* does not lead to immunity, but a gradual clearing of infection with the activation of defence responses.^11^ Indeed in many systems, it has been shown that there is more tolerance than immunity to *V. dahliae*, with the correlation between fungal biomass and disease presence being poor in some cases.^14^

Less is known about the links between pathogen effectors and the mechanism of resistance in other species, however it has previously been reported that, rather than major gene resistance, quantitative resistance is present in many species, including strawberry. Recent work has revealed that multiple effector genes reside in regions of the wilt genome that undergo regular chromosomal reshuffling.^15^ It can therefore be postulated that in the host plant, multiple resistance genes may exist, controlling resistance in a quantitative manner, as has been shown in potato,^16^ though this remains to be formally demonstrated. In *Arabidopsis thaliana*, differences in phenotypic responses to senescence have been linked to differences in tolerance to *V. dahliae*, indicating that resistance responses may be more complex than R gene mediated resistance and that disease ‘escape’ traits may also be important in the plant response to disease.^14^

For most crops *V. dahliae* is controlled in the field by soil fumigation prior to planting. In the EU there is a single remaining soil fumigant, chloropicrin, which is a broad-spectrum treatment for soils and is known to have antimicrobial, herbicide, insecticide and nematicide properties.^17^ Due to the on-going withdrawal of many crop protection agents under Council Directive 91/414/EEC, chloropicrin use in the EU currently faces an uncertain future. There is therefore a need to rapidly develop methods for selecting resistant cultivars of a range of plants in order to ensure alternative strategies for reliable crop production. The mapping progeny of Sargent *et al.*,^18^ developed from the cross ‘Redgauntlet’ × ‘Hapil’ (RG × H), was raised for the purpose of studying the genetic basis of *Verticillium* wilt resistance in the cultivated strawberry. This progeny has been characterized with a large number of microsatellite markers and all 28 linkage groups of the consensus map of the progeny have been characterized, making it a very useful resource for the identification of molecular markers linked to *Verticillium* resistance loci, which would prove invaluable tools for marker-assisted selection in modern *F. ×ananassa*. breeding programmes. We report in this investigation the results of screening the RG × H mapping progeny for resistance to *V. dahliae* in field trials and the identification of QTL associated with resistance.

Following the identification of the QTL, the diploid strawberry reference genome sequence scaffolds (FvH4) underlying the QTL intervals were mined for candidate resistance genes.^19^ These were subsequently mapped onto the octoploid strawberry genome, with varying levels of success. Molecular markers from the candidate genes identified were mapped and validated using cultivated strawberry germplasm of different known resistance statuses. The potential of these markers for marker-assisted selection is discussed.

## Materials and methods

### Field screening

The population, reported previously^18^ consists of a full sib family of 173 individuals generated from a cross between the two strawberry cultivars ‘Redgauntlet’ and ‘Hapil’.

Planting material was cloned by the propagation of runners from mother plants kept in an unheated polytunnel. Plants were established in 9 cm pots from July onwards and then planted in the field in a randomized block design in late Autumn (September–October) of each year for phenotyping in the subsequent year.

Field screening was carried out in three consecutive years with a 10-fold level of replication of all genotypes, in all years. The site was selected on the basis of the fact that it has been a long-established trial site, originally artificially inoculated with a large variety of *V. dahlia*e strains. Disease pressure is maintained from year to year by consecutive plantings of strawberry and also by intercropping between plantings on half of the plot with *Linum usitatissimum* (common flax), a highly wilt-susceptible crop. Wilt counts are taken every year^20^ in order to ensure significant levels of starting inoculum from microsclerotia. In every year, a new field trial was planted in a randomized complete block design with 10 blocks per site, each containing a complete replication of the mapping population and parental genotypes. MyPex^®^ woven groundcover was used to control weed growth and ensure equal spacing between plants.

Data were collected, based upon the above-ground phenotypic manifestation of *V. dahliae* symptoms, ranked on a scale of 1–9, with one being no symptoms and nine being total plant collapse and apparent death.^21^ Data were collected twice per season, at times determined by the development of disease.

### Analysis of field scores

After de-randomisation and grouping of genotypes by accession, data were analysed using R. Mean values of wilt scores across replicates were used for estimating the resistance response. The timepoint at which the histogram of phenotypic values most closely resembled a normal distribution was chosen for QTL analysis, a key assumption of many statistical analyses deployed. Despite these, data was non-normally distributed for most years.

### Linkage analysis and QTL mapping

A linkage map has already been reported for the ‘Redgauntlet × Hapil’ population.^18^ This map was expanded to include additional markers in areas of the genome that were poorly saturated in the previous study. Using the recently published work of Isobe and co-workers^22^ an additional 111 markers were screened within the ‘Redgauntlet × Hapil’ population. Of those, 26 markers were polymorphic and showed segregation in either ‘Redgauntlet’ or ‘Hapil’. These markers (representing a total of 71 loci) were mapped to the previously reported RG × H genetic linkage map.^18^ The new, improved linkage map was used for QTL analysis (Supplementary Tables S1–S3). Despite the additional 71 loci mapped, the linkage map was still poorly saturated on some linkage groups for one or both parents.

Interval mapping was not used due to poor marker saturation of the map and the large number of false positives observed when comparing regions with significant LOD scores with significant regions from preliminary Kruskal–Wallis (KW) testing. As phenotypic data was non-normally distributed, the non-parametric method of KW testing is suitable to identify regions of the genome associated with wilt resistance.^23^ KW analysis identifies markers linked to single-dose QTL and produces a K* statistic, adjusted for ties. KW testing was carried out using MapQTL software.^24^ Markers with a *P* value of 0.005 were selected as those tightly linked to a QTL.

### Bootstrapping

Where reported, confidence intervals were calculated using bootstrapping, as the distribution of phenotypic traits is non-normal. For each bin of progeny members (sizes between 14 and 34), grouped on their marker number, the boot function in *R* was applied with 1000 replicates. Resampling was carried out using the ‘ordinary bootstrap’ method and 95% confidence intervals were calculated with the ‘boot.ci’ function, from the library ‘boot’.^25^

### DNA extraction, PCR, genotyping and analysis

DNA extraction, PCR of SSR markers, analysis on an ABI3130 genetic analyzer and subsequent peak calling, was all carried out as described previously.^18^

## Results

### Multi-year experiments reveal resistance from ‘Redgauntlet’ to be stable

**Figure 1:**
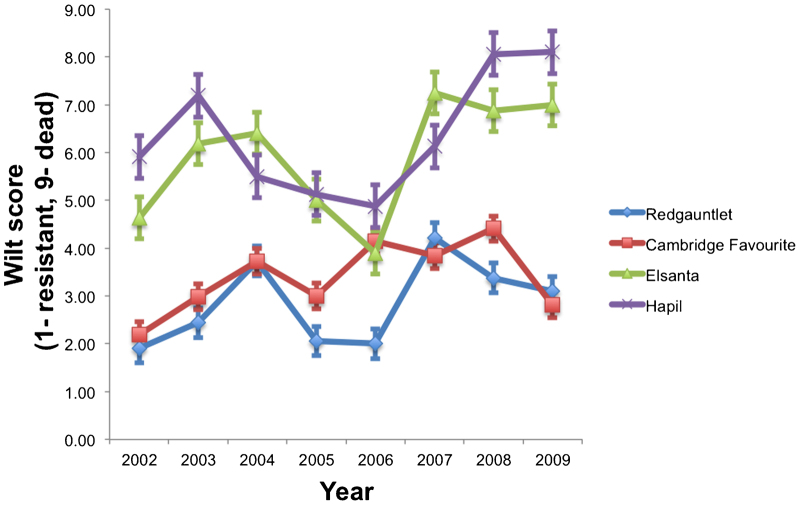
A long-term resistance experiment highlighting year-by-year disease pressure variation for four cultivars, one resistant (‘Redgauntlet’), two of differing degrees of intermediate resistance (‘Cambridge Favourite’, ‘Elsanta’) and one susceptible, (‘Hapil’).

Disease manifestation in the field varies depending upon the season and the climatic variables such as night temperature^26^ as well as genotype. Cultivars with varying degrees of resistance (one highly resistant, one intermediate, two susceptible) were monitored for seven years in an artificially inoculated plot in order to determine if their disease resistance was consistent over multiple years. Each year ten replicates of each plant were planted in a randomized block design. The results show that there are clear seasonal differences in levels of disease development (Figure 1). For example, in 2006 the disease pressure was sufficiently low that the susceptible cultivar ‘Elsanta’ displayed overall wilt scores that were at similar levels to ‘Redgauntlet’ in the following year. Despite the large seasonal variation in 5/7 years ‘Hapil’ was the most susceptible and ‘Redgauntlet’ was the most resistant cultivar. Variance estimates within progeny replicates reveal that variance is not equally distributed among progeny members, with the greatest variance observed in progeny replicates with intermediate wilt scores, indicating either a difficulty in classifying genotypes of intermediate resistance (for example, due to the fact that other developmental traits may be influencing the timing of disease development which are not adequately captured in the phenotyping approach) or a strong genotype by environment interaction when resistance is partial (for example, due to patchy distribution of pathogens with differing virulence across the field) (Supplementary Figure S1).

### Disease development in the field is linear and progressive

**Figure 2:**
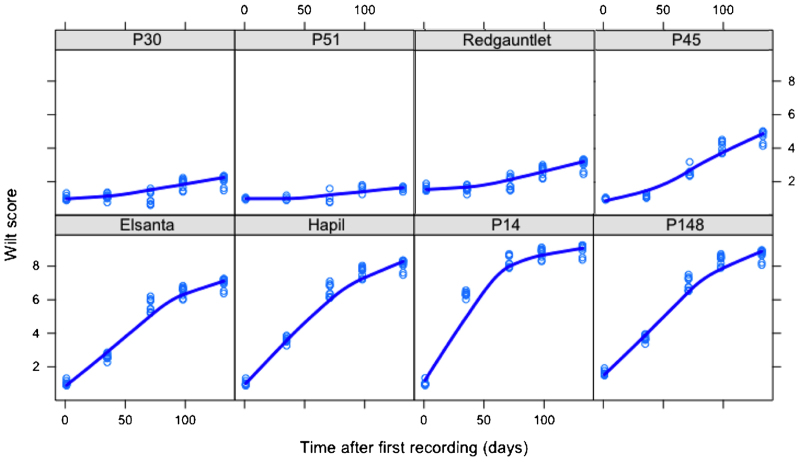
Temporal variation in resistance scores throughout the season in 2009. The *x*-axis depicts the time from the first phenotypic measurement in days (4 July) for eight cultivars and offspring from ‘Redgauntlet’ × ‘Hapil’ (prefixed P), classes as resistant (‘Redgauntlet’, P30, P51) intermediate (P45, ‘Elsanta’) and susceptible (‘Hapil’, P14, P148).

**Figure 3:**
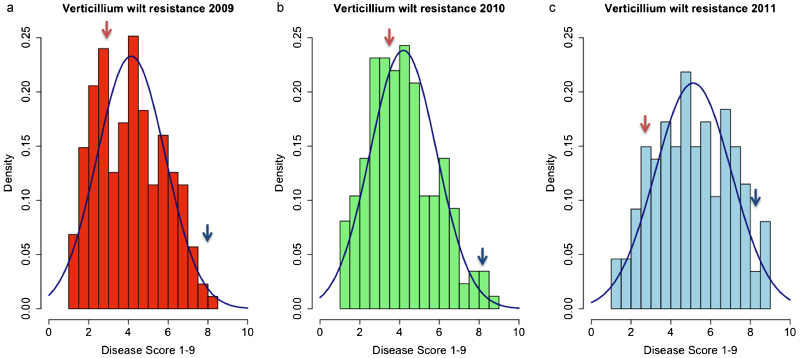
(**a**) A histogram depicting resistance to *Verticillium dahliae* in ‘Redgauntlet’ × ‘Hapil’ progeny in 2009. Overlaid in blue is a normal distribution for the mean and standard deviation of the untransformed phenotypic data. The red arrow indicates the mean phenotypic score of ‘Redgauntlet’, while the blue arrow indicates the mean phenotypic score of ‘Hapil’. (**b**) As **a** but data from 2010. (**c**) As **a** but data from 2011.

The ‘Redgauntlet’ × ‘Hapil’ mapping population was phenotyped in the same way as the long-term resistance experiment, in the field in a randomized block design, with a minimum of six replicates per year. In 2009, the population was phenotyped extensively, along with a standard variety (‘Elsanta’) in order to determine the optimal time for phenotypic evaluation. Figure 2 illustrates the development of above-ground disease symptoms over the season. Comparison of the tails of the mapping population with the two parents and the additional cultivars from the long-term experiment revealed that development of disease symptoms is approximately linear across the season. From the mapping population, observation of the distributions of resistance revealed that the distribution of disease symptoms was most symmetrical and closest to a normal distribution in the two weeks between the end of July and the start of August in all years (Figure 3).

### Disease pressure varies from year to year, but genotypes display similar responses

**Figure 4:**
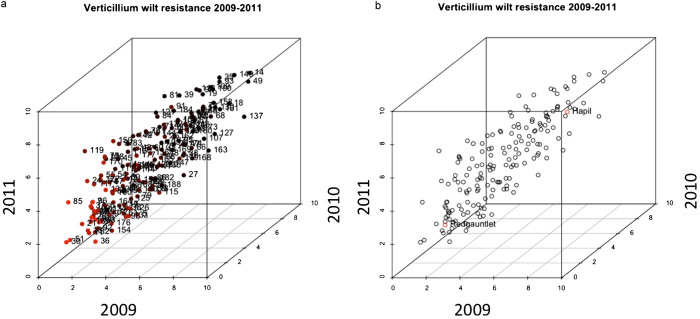
(**a**) Three-dimensional scatter-plot of mean phenotypic scores from three years of field phenotyping (time points selected for QTL analysis) for 173 individuals of the ‘Redgauntlet’ × ‘Hapil’ mapping population. The red colouring indicates increasing resistance. (**b**) As **a**, but highlighting the mean phenotypic scores for the parental lines ‘Redgauntlet’ and ‘Hapil’. Transgressive segregation can be clearly observed across all years.

As observed for the long-term experiment, the disease pressure varied over the three years, with the lowest overall disease pressure observed in 2010. However, the correlation between years was moderately high and always statistically significant (Table 1 and Figure 4a). Again, the variance of the progeny across years varied most for phenotypes with intermediate resistance (see Supplemenary Figure S1 for within year variation). The segregation of resistance in the population is transgressive, with individuals displaying more extreme phenotypes than the parents of the mapping population (Figure 4b; see also Figure 3). Trait segregation was declared transgressive when at least one progeny had a value that was higher or lower than that of the highest or lowest parent, by at least twice the standard deviation of the parents.^27^ The number of transgressive segregant varied between year to year, however two highly resistant individuals and three highly susceptible individuals were significant across all years.

### QTL mapping reveals the oligogenic nature of resistance

**Figure 5:**
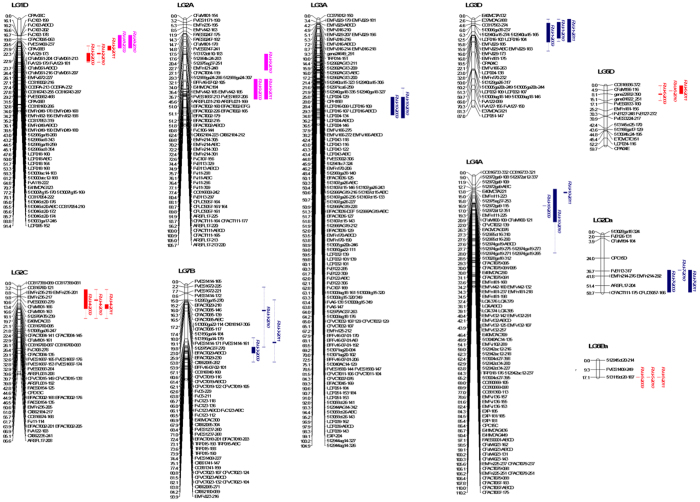
Combined map of ‘Redgauntlet’ × ‘Hapil’, depicting the position of *Verticillium dahliae* resistance QTL. Markers with a significance level of *P*<0.005 are highlighted in boxes, with p values between *P*<0.005 and *P*<0.05 shown as whiskers. Red boxes are QTL from ‘Redgauntlet’, blue boxes are QTL from ‘Hapil’ and pink are QTL found in both cultivars.

Using data from the three years, QTL were mapped treating each year separately, using KW testing. Resistance was detected at seven loci across all three years, with a further locus significant in two out of three years and three loci highly significant but only in a single year (Table 2 and Figure 5). This led to a total of 11 QTL loci that were identified in at least one year of analysis.

### Comparison of QTL effects across years

**Figure 6:**
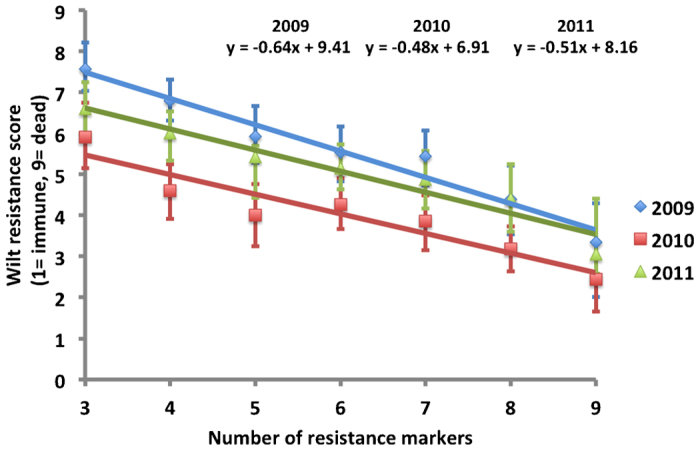
Plot of the relationship between mean wilt score of individuals binned by marker number (error bars are 95% confidence intervals), for phenotypic data of three years (minimum of ten individuals per bin). A clear and consistent increase in wilt resistance is seen across all years with increasing marker number. However, the level of wilt resistance observed depends upon the disease pressure for that year. 2009 was the most severe year for disease, followed by 2011 and then 2010. For comparison mean wilt scores of ‘Redgauntlet’ and ‘Hapil’ in 2009 were 3.1 and 8.1, respectively). Linear regression analysis of the relationship was performed and the gradient and intercept are shown for each year.

In order to understand whether there were effects of QTL significant in only one year or across multiple years, an analysis was carried out where the mapping population was binned into groups containing up to two markers, three markers, all the way to nine markers, ensuring a minimum of ten genotypes per bin. Markers for which the presence allele was in coupling with the QTL were selected, as these were the only markers considered suitable for validation of QTL in the wider germplasm. In several cases (those marked with a suffix M1 in Table 2) these were not the most strongly associated marker with the QTL. 95% confidence intervals were calculated for the average phenotypic wilt scores in each bin by non-parametric bootstrapping within binned groups of individuals. A clear linear decline in disease susceptibility was observed with the increase in number of wilt markers, per genotypic bin (Figure 6). This was observed across all years, with the gradient of the slope and the intercept on the *y* axis both associated with the level of disease pressure in that year. Hence, the slope was steepest in 2009, then 2011 and last 2010, which was the year with the lowest disease pressure.

### Validation of markers in other germplasm

Effective translation of markers into breeding material is important if QTL are to be deployed in a breeding programme. Using historic trial data from the UK national strawberry breeding programme at East Malling Research and literature surveys, germplasm was binned into four groups; Reported Resistant (RR), a class where no trial data at East Malling Research exists; Resistant (R), a class where resistance has been shown in trials (in the same infested plot); Intermediate (I), where disease progress is variable and intermediate symptoms sometimes develop; Susceptible (S), where material is consistently susceptible to wilt.

Considering each marker in isolation revealed that in four cases, *RVd5, RVd6, RVd9* (‘Redgauntlet’ allele) and *RVd11* there was a significant enrichment (*P*<0.05, two tailed *t*-test with unequal samples sizes and unequal variance) of alleles in the resistant set. There was no significant enrichment of marker frequency in the resistant set *versus* the intermediate and susceptible sets (*t*-tests with unequal sample sizes and unequal variance) in nine out of the thirteen cases (although in five of these cases the allele frequency was higher in the resistant set).

When considered cumulatively, the average number of markers in each class increased in accordance with increasing resistance of the cultivar in the following manner: ‘RR’ and ‘R’ classes had an average of six markers per cultivar, ‘I’ class had an average of five markers and ‘S’ class had an average of four markers. There was a clear and statistically significant difference between the total number of resistance markers found in RR and R cultivars *versus* I and S (mean: 4) (*P*<7.7×10^−5^, two tailed *t*-test with equal sample sizes and equal variance F-test 0.999) or between RR and R cultivars and S cultivars (*P*<3.52×10^−5^, two tailed *t*-test with unequal sample sizes and equal variance, *F*-test 0.999). Despite the clear overall trend there were some anomalous results, for example, some resistant lines (e.g., EM0555) had only three markers, while other susceptible lines (e.g., EM1871) had six markers. However, it should be noted that ‘Hapil’ itself contained six resistance markers (four present only in ‘Hapil’, and two shared with ‘Redgauntlet’).

## Discussion

Resistance to *Verticillium* wilt is a trait that is under complex control in strawberry and there are multiple QTL that act to confer resistance. Field screening appears to be an effective and robust method of differentiating individuals based upon a quantitative measure of their disease resistance. The data presented in this paper clearly show that the larger the number of resistance QTL that an individual harbours, the greater the chance of field resistance or tolerance. Resistance from any single QTL is of small effect and QTL appear to act (in a field setting) in an additive manner. This does not preclude there being non-additive effects between loci (i.e., gene for gene resistance) between different isolates of *Verticillium* wilt and different host genotypes; however, the current data do not permit testing for such interactions, as this would require the use of single spore cultures and controlled inoculations.

It is apparent that there are instances where multiple homoeologues contain QTL. *RVd4, RVd7* and *RVd9* all contain QTL present in homoeology group 2 while *RVd1* and *RVd10* are present on chromosomes in homoeology group 3. The evidence for homoeo-QTL is currently weak, as significant QTL regions on homeologous chromosomes currently have no homoeologue-spanning SSR markers in regions of high significance. This does not preclude the presence of homoeo-QTL, simply that with the current low-resolution SSR linkage map, there is a lack of power to identify homoeo-QTL.

Pyramiding so many markers (perhaps between eight or nine) to ensure resistance requires careful design of crosses within a breeding programme.^28^ However, the data presented in this study (Table 3) suggest that pyramiding resistance markers should be a relatively straightforward task. For example, a cross between EM1624 (six markers) and ‘Albion’ (seven markers) would lead to a progeny segregating for 11 out of a total of 13 possible resistance markers (there are more markers than QTL due to shared QTL having different allele sizes associated with each QTL in the most closely linked marker). Only two markers are common between the cultivars, indicating that it should be possible to pyramid markers effectively. Assuming all markers are heterozygous in a progeny of 1000, 837 of the progeny should contain at least six markers, while 350 should contain at least nine markers, i.e., approximately one in three. Thus, even simple parental selection using the markers greatly improves the probability of combining multiple resistance markers in a progeny compared to using empirical selection.

The fact that there are cultivars in the resistant set that have fewer resistance markers than either ‘Redgauntlet’ or ‘Hapil’ present a number of possible hypotheses. First, that in some genotypes the linkage between the chosen markers (which in some cases have already been shown not to be the closest linked to the QTL) is incomplete. Second, that linkage disequilibrium between the QTL marker and the QTL of interest is incomplete due to the fact that the marker under consideration is either a younger or older mutation than the focal QTL and therefore is only partially associated with the focal QTL. Third, that there are multiple resistance sources for *Verticillium* and the biallelic cross of ‘Redgauntlet’ and ‘Hapil’ only contains a portion of the resistance that is present in the wider strawberry germplasm. All of these hypotheses are equally valid. To address these hypotheses, it will be necessary to improve the level of marker saturation. Despite the large amount of effort already deployed in developing SSR markers, the ‘Redgauntlet’ × ‘Hapil’ population still has poor coverage of some areas of the genome, with fewer than five markers on some linkage groups. The high degree of homozygosity observed in *F. ×ananassa* genotypes used as parents for linkage map development^18,29^ may mean that certain genomic regions cannot be saturated with any type of molecular marker on maps developed using those IStraw90 genotypes as parents. Improved marker saturation can be accomplished with a genotyping platform such as the recently described IStraw90 SNP chip. However, as in many other cases,^30^ the small number of samples used to call genotypes may hamper population level studies. The use of an ‘open’ rather than a ‘closed’ genotyping service (such as a sequencing based genotyping approach) may minimise the likelihood of this phenomenon occurring in any future association genetics studies.^31^ However, the large founder effect thought to be present in a lot of strawberry germplasm, may mean that there are a limited number of haplotypes in the strawberry genepool and therefore ascertainment biases are likely to be a lesser problem than in human studies.

Any association genetics approach would also need to be coupled with a greater understanding of how heterogeneous the pathogen population is. In future studies, it would be desirable to conduct the QTL analysis with a number of clearly defined isolates of differing virulence in order to identify how isolate virulence performs against host resistance. Furthermore, analysis of additional QTL in the ‘Redgauntlet’ × ‘Hapil’ population could reveal whether QTL are pleiotropic.

## Figures and Tables

**Table 1:** Correlation between years (Spearman rank) and significant for phenotypic data

	2009	2010	2011
2009	X	x	x
2010	0.68 (*P*<2.2×10^−16^)	x	x
2011	0.711 (*P*<2.2×10^−16^)	0.64 (*P*<2.2×10^−16^)	x

**Table 2:** Resistance QTL and validity across years using the most closely linked markers (M1 are not most closely linked markers) but are the closest markers in coupling to the QTL

						*K*		
QTL	Linkage group	Position (cM)	Years valid	Phase correct	Parent	2009	2010	2011
*RVd1-M1*	3D	16.75	3	N	Hapil	10.147	15.847	8.075
*RVd2*	1D	27.41	3	Y	Redgauntlet	14.706	6.6	3.758
*RVd3*	7B	62.63	3	Y	Hapil	8.441	5.057	7.074
*RVd4-M1*	2C	3.17	3	N	Redgauntlet	10.169	4.238	12.418
*RVd5*	6B	17.08	3	Y	Redgauntlet	18.478	5.208	16.988
*RVd6-M1*	5D	8.13	1	N	Redgauntlet	7.113	3.063	4.713
*RVd7*	2D-A	58.70	3	Y	Hapil	5.933	2.855	8.645
*RVd8*	1D	16.22	1	Y	Both	18.443	5.8	4.761
*RVd9-M1*	2A	76.14	2	N/Y	Both	13.507	0	7.133
*RVd10*	3A	93.30	3	Y	Hapil	5.64	13.476	3.424
*RVd11*	4A	19.32	1	Y	Hapil	0.734	6.681	3.033

**Table 3:** Validation of QTL in wider germplasm using most closely linked in-phase markers A number 1 indicates marker presence (RR, reported resistance; R, resistant (EMR test); I, intermediate; S, susceptible)

QTL		*RVd1*	*RVd2*	*RVd3*	*RVd4*	*RVd5*	*RVd6*	*RVd7*	*RVd8*	*RVd8*	*RVd9*	*RVd9*	*RVd10*	*RVd11*
	Parental Origin (Allele below)	H	R	H	R	R	R	R	R	H	H	R	H	H
Phenotype	Cultivar	276	213	231	166	187	252	169	202	199	309	327	342	225
RR	Evie2			1		1	1	1		1		1		1
RR	Merton Dawn						1	1				1		1
RR	Sierra		1		1	1	1	1				1		1
RR	Wiltguard						1			1	1			1
R	Alice			1		1	1	1						1
R	Cupid (EM1395)	1		1	1	1	1			1	1		1	1
R	EM0555		1			1		1						
R	EM0701	1		1	1	1	1			1				1
R	EM0972			1	1				1					1
R	EM1677			1		1			1					1
R	EM1785			1		1	1			1				1
R	Fenella (EM1308)	1		1	1	1	1		1		1			
R	Flamenco			1		1	1	1					1	1
R	Florence			1		1	1		1					1
R	Judibell		1	1	1	1	1		1					1
R	Pegasus			1			1	1	1			1		1
R	Senga Sengana	1		1		1	1				1			1
R	**Redgauntlet**		**1**		**1**	**1**	**1**	**1**	**1**			**1**		**1**
R	EM1682			1	1	1		1	1					1
R	EM346 (finesse)			1		1	1			1	1	1		1
R	EM1624		1	1			1				1		1	1
R	Albion	1		1		1		1		1		1		1
R	Everest			1		1	1			1		1		1
R	Sonata	1		1				1	1		1		1	1
I	Cambridge Favourite		1			1		1	1		1		1	
I	EM1727						1			1				1
I	EM1792		1			1	1	1	1					1
I	EM1399	1		1			1			1				1
I	EM1500			1	1	1	1	1	1					1
I	Elegance			1		1			1					1
I	Malling Pearl			1		1			1			1	1	
I	Symphony					1		1	1					
S	EM1442					1	1			1			1	1
S	EM1580									1			1	1
S	EM1733						1	1	1					1
S	EM1764					1	1	1		1				
S	EM1772	1		1						1				
S	EM1812	1		1		1			1					1
S	EM1871			1			1		1		1		1	1
S	Holiday			1	1									
S	Mae	1		1						1				1
S	Vibrant	1		1		1			1					1
S	**Hapil**	**1**		**1**						**1**	**1**		**1**	**1**
S	Emily				1				1		1			
S	EM1856					1								1
S	EM1942			1						1				
S	EM1966			1						1				
S	Elsanta			1				1	1		1		1	1
S	Eros			1				1	1					1
