# Supplementary material for: Mapping QTL associated with Verticillium dahliae resistance in the cultivated strawberry (Fragaria × ananassa)
Source: Hortic Res. 2015 Mar 11;2:15009–. doi: 10.1038/hortres.2015.9 (PMC4595976; doi:10.1038/hortres.2015.9)
Supplement: Supplementary File [file hortres20159-s1.docx]

**Supplementary table 1-** Number of markers mapped on combined map for ‘Redgauntlet’ × ‘Hapil’

| Linkage Group | Rg × H | | | | | | | | |
| --- | --- | --- | --- | --- | --- | --- | --- | --- | --- |
|  | AB × BB | AA × AB | AB × AB | SSRs | AFLPs | Total Markers | Total Loci | Length (cM) |  |
| LG1-A | 23 | 11 | 11 | 18 | 8 | 26 | 45 | 112 |  |
| LG1-B | 23 | 28 | 36 | 30 | 4 | 34 | 87 | 78 |  |
| LG1-C | 20 | 14 | 9 | 18 | 3 | 21 | 43 | 85 |  |
| LG1-D | 24 | 21 | 10 | 24 | 4 | 28 | 55 | 91 |  |
| LG2-A | 20 | 15 | 22 | 25 | 2 | 27 | 57 | 106 |  |
| LG2-B | 13 | 12 | 9 | 17 | 3 | 20 | 34 | 98 |  |
| LG2-C | 16 | 20 | 7 | 20 | 2 | 22 | 43 | 87 |  |
| LG2-Da | 10 |  |  | 8 | 1 | 9 | 10 | 59 |  |
| LG2-Db |  | 6 |  | 6 |  | 6 | 6 | 52 |  |
| LG3-A | 23 | 36 | 49 | 42 | 3 | 45 | 108 | 105 |  |
| LG3-B | 17 | 13 | 9 | 21 | 4 | 25 | 39 | 110 |  |
| LG3-C | 13 | 14 | 14 | 17 | 3 | 20 | 41 | 121 |  |
| LG3-D | 1 | 26 | 2 | 16 | 3 | 19 | 29 | 87 |  |
| LG4-A | 32 | 23 | 27 | 27 | 8 | 35 | 82 | 110 |  |
| LG4-B | 1 | 9 | 7 | 11 | 1 | 12 | 17 | 53 |  |
| LG4-C | 10 | 6 | 5 | 10 | 4 | 14 | 21 | 86 |  |
| LG4-D | 16 | 5 | 2 | 15 | 2 | 17 | 23 | 112 |  |
| LG5-A | 7 | 8 | 4 | 9 | 4 | 13 | 19 | 37 |  |
| LG5-B | 13 | 15 | 17 | 19 | 3 | 22 | 45 | 87 |  |
| LG5-C | 4 | 7 | 14 | 11 | 1 | 12 | 25 | 44 |  |
| LG5-D | 13 |  | 2 | 12 | 2 | 14 | 15 | 60 |  |
| LG6-A | 10 | 26 | 26 | 25 | 10 | 35 | 62 | 155 |  |
| LG6-Ba | 1 |  | 2 | 3 |  | 3 | 3 | 17 |  |
| LG6-Bb | 5 | 7 | 7 | 8 | 3 | 11 | 19 | 52 |  |
| LG6-C | 11 | 1 | 10 | 10 | 6 | 16 | 22 | 140 |  |
| LG6-D |  | 15 |  | 10 | 3 | 13 | 15 | 115 |  |
| LG7-A | 5 | 10 | 6 | 12 | 1 | 13 | 21 | 53 |  |
| LG7-B | 19 | 19 | 16 | 23 | 2 | 25 | 54 | 94 |  |
| LG7-C | 2 | 8 | 8 | 9 | 2 | 11 | 18 | 39 |  |
| LG7-D | 22 | 21 | 26 | 26 | 4 | 30 | 69 | 113 |  |
| Unmapped-1 | 3 |  |  | 2 | 1 | 3 | 3 | 11 |  |
| Unmapped-2 | 3 |  |  | 3 |  | 3 | 3 | 18 |  |
| Total | 380 | 396 | 357 | 507 | 97 | 604 | 1,133 | 2,586 |  |

**Supplementary Table 2-** Additional markers mapped onto ‘Redgauntlet’ × ‘Hapil’ in this study

| Linkage Group | 26 Novel Markers | |
| --- | --- | --- |
|  | Markers | Total Loci |
| LG1-A | 1 | 1 |
| LG1-B |  |  |
| LG1-C |  |  |
| LG1-D | 2 | 2 |
| LG2-A | 4 | 6 |
| LG2-B | 2 | 3 |
| LG2-C | 2 | 8 |
| LG2-Da |  |  |
| LG2-Db | 2 | 2 |
| LG3-A | 2 | 3 |
| LG3-B | 1 | 1 |
| LG3-C | 3 | 3 |
| LG3-D |  |  |
| LG4-A | 1 | 1 |
| LG4-B | 1 | 4 |
| LG4-C | 1 | 1 |
| LG4-D |  |  |
| LG5-A | 1 | 1 |
| LG5-B | 3 | 4 |
| LG5-C | 1 | 3 |
| LG5-D | 2 | 2 |
| LG6-A | 3 | 4 |
| LG6-Ba | 1 | 1 |
| LG6-Bb |  |  |
| LG6-C | 1 | 1 |
| LG6-D |  |  |
| LG7-A | 3 | 4 |
| LG7-B | 4 | 9 |
| LG7-C | 1 | 1 |
| LG7-D | 4 | 6 |
| Unmapped-1 |  |  |
| Unmapped-2 |  |  |
| Total | 46 | 71 |

**Supplementary Table 3-** Additional markers on the ‘Redgauntlet x Hapil’ map

|  | Isobe et al. 2013 | | This study | |
| --- | --- | --- | --- | --- |
| Name | Forward sequence | Reverse sequence | LG | Loci Mapped |
| FVES0982 | tcttcaaagccacaaccctc | gatctcattgcatgcttaggg | LG1A, LG1D | 1,1 |
| FAES0154 | cgccaaaacttggtagatgg | atcaggcaccaattgacctc | LG2C | 2 |
| FVES1687 | acgaagggtgaagggtctct | cccaaaaacccaaatcctct | LG2A, LG2C, LG2Db | 2,4,1 |
| FAES0247 | acgccttcgatccttttctt | caaggcagtaaagctcccag | LG2A, LG2B, LG5B | 2,1,1 |
| FVES0347 | aactcctcctcctcctcgtc | gtaaggagcagagccactcg | LG2A | 1 |
| FVES0393 | aagccatctcattcaccgat | gcgacaaaggcaagaatagc | LG2B, LG2C | 2,2 |
| FVES0936 | cacatatataaaccccagtcgg | tgaaggaagggatggagttg | LG2Db | 1 |
| FVES1171 | tgaatacatgcatcgctggt | gtgggaaacaaagtctggga | LG2A, LG3B | 1,1 |
| FVES3374 | agttcctcccttctcgcttc | gttgatgtagctgtacgccg | LG3C | 1 |
| FVES3002 | actcggagtaggaaatgcca | ctgttgatggtggtagggct | LG3A | 1 |
| FVES3364 | gaccaccgccactctctaaa | ggtgttgaggaaggcgtaga | LG3C | 1 |
| FAES0001 | gggctcaaaagatgtggaaa | tttatttgggaaggcatcgt | LG4A, LG4C | 1,1 |
| FAES0063 | aacccagatgaaattgctgc | cccagtgacaaacaagcaga | LG4B | 4 |
| FVES1409 | tcggtttctcgctttctttc | gtgatccgatacgttggctt | LG1D, LG6A, LG6Ba, LG7B, LG7D | 1,1,1,1,1 |
| FVES0545 | gcaagtccatatccacttctttg | tcaaattccgtttcgtcctc | LG6A | 1 |
| FVES3224 | taacttccctcccgattcct | cctcttgaagctccgatcac | LG5B, LG5D | 2,1 |
| FVES0833 | agccaagaagccagaagaca | cctgctctcgtcatccattt | LG5B, LG5D | 1,1 |
| FAES0382 | ataccagaacccaccaccaa | gtggttcccagagctgaaag | LG5A, LG5C | 1,3 |
| FVES0013 | tctcctcctctcttcccgat | gaaatgctctcttcggttcg | LG3C, LG6A | 1,2 |
| FATS0090 | agagccggtttagctgagtg | cgtcgtcgttttctctcctc | LG6C | 1 |
| FVES1580 | taaacgacatcggcgacata | agaatcagatggtgttgccc | LG3A | 2 |
| FATS0076 | caagggaagtggaagtggaa | gctgaggagaaacctggaga | LG7A | 1 |
| FVES1672 | acaccctgtcccttcacaag | gaaagatgacttcggcttcg | LG7A, LG7B, LG7C, LG7D | 1,2,1,2 |
| FVES1237 | gtgtcactcacacacacacca | caccttctccattccctgag | LG7B | 2 |
| FVES1414 | atctcgaggcttccaagaca | aatcgggatattcgcattaca | LG7A, LG7B, LG7D | 2,4,1 |
| FVES1834 | gttgaagcagctcccaaaag | gaattgacgaggcggtaaaa | LG7D | 2 |
| Total |  |  |  |  |
| 26 |  |  | **46** | **71** |


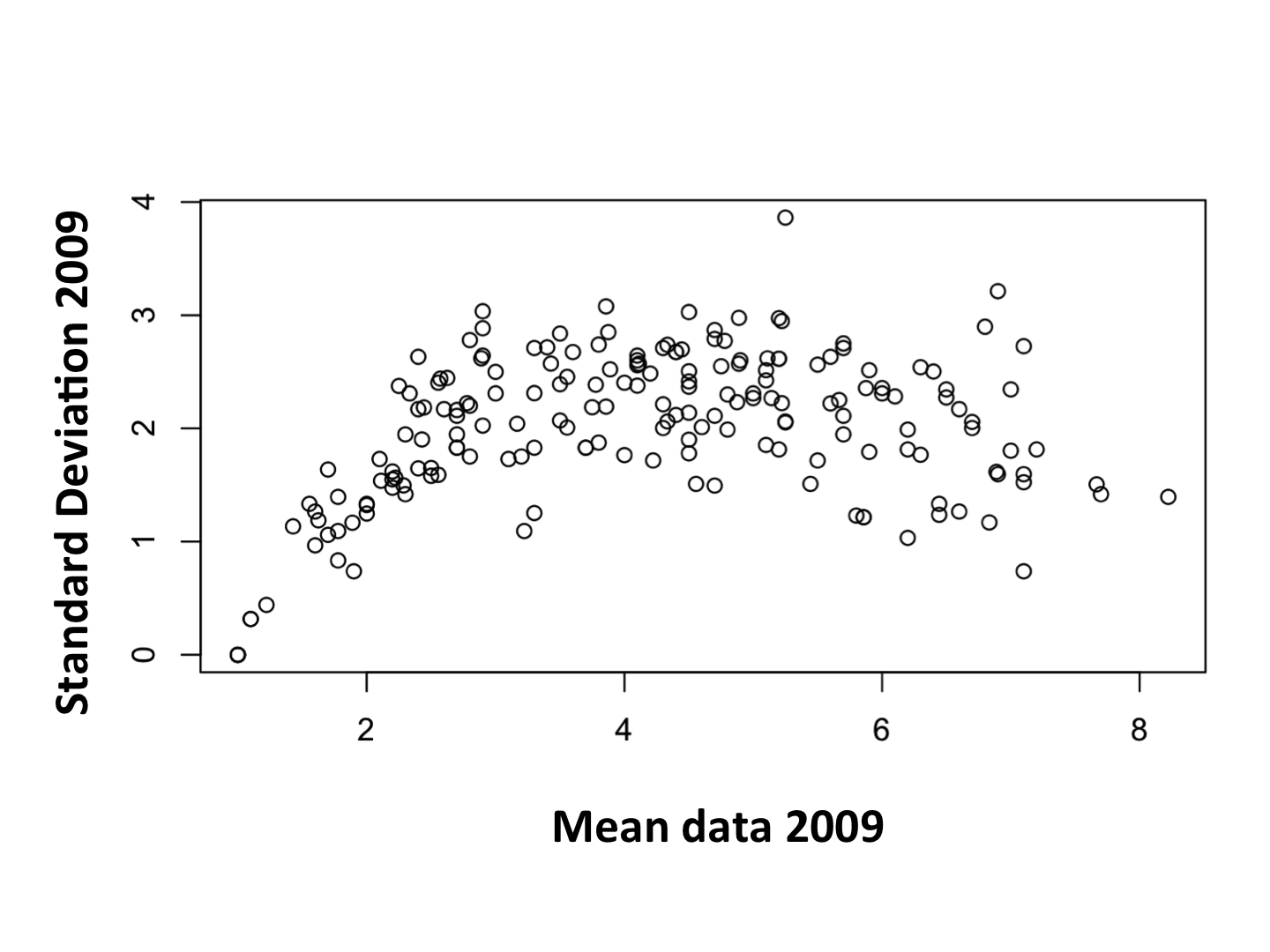


Supplementary Figure 1
